# Explore factors influencing residents' green lifestyle: evidence from the Chinese General Social Survey data

**DOI:** 10.3389/fpubh.2025.1527247

**Published:** 2025-02-19

**Authors:** Yifei Li, Jiyue Li, Chuntian Lu

**Affiliations:** Institute for Empirical Social Science Research, Xi'an Jiaotong University, Xi'an, Shaanxi, China

**Keywords:** green lifestyle, drop-off recycling, low-carbon purchasing, civic advocating, environmental protection intention

## Abstract

**Introduction:**

This paper explores the multifaceted dynamics and various determinants impacting residents' green lifestyles in China, focusing on drop-off recycling, low-carbon purchasing, and civic advocating.

**Methods:**

Using data from the 2021 Chinese General Social Survey and the Chinese Statistical Yearbook, we provide an integrated paradigm to position green lifestyles in a hierarchical framework, explore the individual-level mediating mechanisms nested within macro influences, and elucidate the relative strengths of determinants.

**Results and discussion:**

Firstly, the random coefficient regression models show that environmental knowledge and environmental protection intention significantly positively affect green lifestyles. Adopting traditional media could enhance civic advocating, and using new media may bolster low-carbon purchasing tendencies. A higher per capita GDP, increased public expenditure on environmental protection, and a reduced proportion of secondary industry are significant factors that promote drop-off recycling and low-carbon purchasing. Secondly, the multilevel mediational effects identify that people's environmental protection intention is a salient mediating variable within the control of regional macro factors. Additionally, the sheaf coefficient clustering models underscore the prominence of ecological awareness and macro-level factors in shaping drop-off recycling and civic advocating.

## 1 Introduction

The global environment underwent a large-scale and rapid transformation with the widespread use of fossil fuels, urban expansion, rapid population growth, and the spirit of conquering nature, which have given rise to a series of ecological and environmental problems since the Industrial Revolution (1, 2). A bulk of research has analyzed the immediate and long-term consequences of environmental pollution, ecological degradation, and climate change on mental and physical health (3, 4). This has led to a call for the universal adoption of environmentally friendly behavior and green lifestyles (GL) (5, 6).

GL is defined as a pattern of living that contains thoughts about the uncertain environmental impacts of daily practices and corresponding personal reflections and adaptions (7). It refers to a combination of conservation-oriented behaviors and sustained participation performed by individuals spontaneously and consciously for pro-environmental reasons (8, 9). GL has abundant environmental and sociological connotations. On the one hand, GL implies one's perception of social status, constituting a facet of identity and self-expression in a specific context, delineated by social class and access to informational resources. On the other hand, GL shows people's subjective proactivity to cope with ecological insecurity, which means that people do not merely adopt isolated and fragmented behavioral decisions but aspire toward multiple systematizations within the spheres of family, occupation, community, online interactions (7, 10). This conceptualization bears significant theoretical and practical implications. The exploration of GL should not be confined to the existing utilitarian perspectives centered around acquiring commodities and services (11); instead, it should be expanded to encompass the ongoing processes.

Previous studies analyze the factors influencing GL from multiple aspects. A meta-analysis of 54 empirical studies organized three categories that pose an impact on green purchase intention, including cognitive factors, consumer characteristics, and social factors (12). Similarly, a systematic review of 151 empirical studies identified that individual concerns, cultural norms, political factors, psychographic traits, ethical values, and product-related variables were significantly correlated with green purchase behaviors and intentions (13). These studies provide foundational variables for understanding GL with a particular focus on the consumption dimension. While important, green consumption represents only one aspect of GL. Other critical components, such as low-barrier everyday environmental behaviors (e.g., recycling or energy-saving habits) and higher-demand civic green political participation (e.g., advocacy or policy engagement), also warrant analysis and discussion to fully capture the multidimensional nature of GL.

Citizens of China show a regrettable inadequacy in environmental knowledge, consciousness, and behaviors, revealing a conspicuous reliance on government interventions to reduce environmental degradation, which is often described as “government-oriented-dependent” (14). However, the government usually lags in mitigating ecological challenges (15). Chinese residents are encouraged to develop and embrace a sustainable and ecologically GL to foster and mobilize public engagement, but they encounter challenges including underdevelopment of green markets, limited availability of green products, poorly constructed resource recycling systems, and barriers to achieving green transformation (16). Studies about single domains of GL aim to analyze how individuals participate in “4 Rs” activities (reducing, reusing, recycling, and purchasing), the extent to which they participate, and their following impacts and group differences (17, 18).

Currently, empirical work in this area predominantly concentrates on advanced industrialized nations and regions (19), leaving a considerable gap in the literature concerning the examination of GL among Chinese citizens. The country offers a social context of collectivism, socialist cultural values and ethics, and government-led environmental management (17). Also, the narrow focus in the previous studies of single pro-environmental behaviors usually implies limited beneficial impact (9), so an organizing taxonomy of social, economic, and political green actions could be applied to explain the incorporation of past/current choices and future decisions.

This paper delves into the contemporary landscape and the various determinants impacting China's GL and contributes to green behavior studies in three ways. First, we adopt an integrated paradigm to position GL in a hierarchical framework with multiple domains. The framework allowed for exploring an individual's engagement in a spectrum of green behaviors, ranging from elementary to intricate, to form a process of gradual life change. Second, we analyzed the multilevel influences stemming from micro and macro factors that shape GL, explored the individual-level mediating mechanisms nested within macro influences, and elucidated the relative strengths of different determinants. It provided empirical support for understanding GL's group differentiation and influence mechanisms. Third, we extended the research scope to the specific context of China, highlighting the regional differences in the governmental role of environmental protection. We also tried to offer practical insights for policymakers seeking to promote sustainable, responsible behavior among Chinese citizens.

## 2 Literature background and conceptual framework

The environmental psychological and sociological paradigms have evolved, developed, and amalgamated from them in response to the increasing attention to prominent green behaviors and lifestyles.

### 2.1 The environmental psychological paradigm

The environmental psychological paradigm examines how individual-level psychological factors influence environmental behaviors. The following three representative theories progress from the generation of environmental awareness, to the development of behavioral patterns, and finally to the activation of specific actions, highlighting psychological drivers and normative influence on individuals' green behaviors.

*The norm activation theory* aims to predict whether personal normative beliefs will motivate individuals to participate in pro-social or altruistic behaviors (20). These normative beliefs often stem from personal education and relevant knowledge, activated by awareness of behavioral consequences and assumed responsibility, serving as the basis for shaping their values to altruism (21). Current investigations concentrate on elements, such as environmental knowledge, self-regulatory emotions, and environmental grievances, in shaping attitudes about green consumption (22–25).

*The value-belief-norm theory* starts from an individual's general code of conduct to lead to the formation of beliefs, which enable people to perceive the threat posed by the possible adverse behavioral outcomes, further evolving into a sense of environmental responsibility and specific eco-friendly behaviors (26–29). Political consciousness is a factor of interest in this theory, as individuals with liberal political values often exhibit stronger intentions to engage in green behavior and environmental concerns than conservatives (30).

*The theory of planned behavior* posits that behavioral intentions for various actions could be predicted by three key factors: attitude toward the behavior, subjective aspiration, and perceived behavioral control (31). People usually choose a goal and make plans on a continuum from intrinsic to extrinsic, influencing the actual behaviors (32, 33). A two-wave study of Italian workers demonstrated a strong association between intentions and green energy-saving behaviors (34).

We extend the above theories from explaining single environmental behaviors to encompassing an overall GL, and propose the following hypotheses:

**Hypothesis 1.1**: Personal environmental knowledge has a positive relationship with GL.

**Hypothesis 1.2**: Personal environmental protection intention has a positive relationship with GL.

**Hypothesis 1.3**: Personal environmental risk perception has a positive relationship with GL.

Nevertheless, research at the individual level faces criticism of the inconsistency between attitudes and behaviors that people may have positive intentions to recycle, but only a few of them would adopt green practices (35, 36). It is essential to consider the external influences of social factors to gain a more comprehensive understanding of GL.

### 2.2 The environmental sociological paradigm

The environmental sociological paradigm transcends the constraints of individual psychological components, focusing on the external influences of the social environment. Two widely-discussed hypotheses are grounded in the framework of social constructivism, with shared focus on human-environment dynamics and the divergent consequences of social development.

*Constructivism* believes that an environmental phenomenon turns into a social problem when it is constructed by society and accepted by the public through propaganda and social media (37). Especially for Generation Z, the Internet and social media can be leveraged to garner interest and spread information about leading GL, such as accommodating green behavior, recycling, cutting back on electricity use, and eliminating paper use in the workplace (38–40). However, sometimes social media was ineffective in bringing about large-scale behavioral change (41), so, the role of conventional media is still worthy of observation.

*The prosperity hypothesis of economic development* suggests that in societies with higher economic prosperity, the public tends to have greater environmental awareness and engagement in green practices (42). Sixty six studies from 28 countries discovered that in industrialized countries, the intention to behave environmentally was more likely to translate into actual conduct to lead a sustainable lifestyle (43). But some studies also indicate that compared to the citizens of impoverished countries, those in prosperous nations may even be less likely to engage in green consumption and environmental conservation actions due to tax concerns and moral inclination (44, 45).

*The driver hypothesis of environmental pollution* contends that individual environmental behaviors are determined by objective environmental conditions and adaptive actions (46, 47). The more adverse the objective conditions, the more people are awakened to environmental consciousness and behaviors reacting to pollution stimuli and cues (48). The impact was found to be limited, considering the varied environmental knowledge of the public, the imperceptible contaminations, and the national political conditions (49, 50).

We propose the following hypotheses to test the macro-level factors' correlations with residents' GL:

**Hypothesis 2.1**: Regional economic development has a positive relationship with GL.

**Hypothesis 2.2**: Regional pollution conditions have a positive relationship with GL.

**Hypothesis 2.3**: Regional environmental governance expenditures have a positive relationship with GL.

### 2.3 The integrated paradigm in the current research

This paper integrates the environmental psychological and sociological paradigm to address the research gaps identified in the existing literature. We proposed three critical extensions:

First, previous studies on environmental behavior have been frequently separated into the private and public spheres (51, 52) and environmental activism vs. conservation actions (36, 53), or just treats GL as single environmental behaviors, emphasizing consumption, transportation, and energy-saving behaviors. This paper emphasizes the holistic nature of individual engagement in environmental actions by merging environmental behavior into the context of GL. The range comprises low-barrier-to-entry activities such as drop-off recycling, subjectively motivated and decided low-carbon purchases, and civic advocacy that demands a particular degree of self-cultivation achievement.

Second, environmental threats often have latency and imperceptibility when situated within the framework of a risk society (54). The ongoing social changes make it hard to examine the underlying mechanisms of GL. Previous studies have insights in the translation from environmental knowledge to pro-environmental behaviors and found mediating factors of behavioral intentions (18), environmental values (55), and green commitment (56). However, most of these mechanism studies primarily focus on the impact limited to the individual level. This study builds on the theory of planned behavior and takes a step further to explore the pathways and efficacy of individual-level mechanisms structured within macro-level factors. We propose the following hypothesis:

**Hypothesis 3**: Nested within the macro structural influence, personal environmental protection intention mediates the relationship between environmental knowledge and GL.

Third, in light of China's societal transformation, we focus on the role played by government initiatives in improving environmental quality, combating pollution, and encouraging resource conservation. Influenced by the social system and fiscal structure, disparities exist in the public expenditures on environmental protection between central and local governments in different regions (57). Incorporating government management and the protection hypothesis of public governance into the framework can highlight regional differences in China's environmental management.

**Figure 1:**
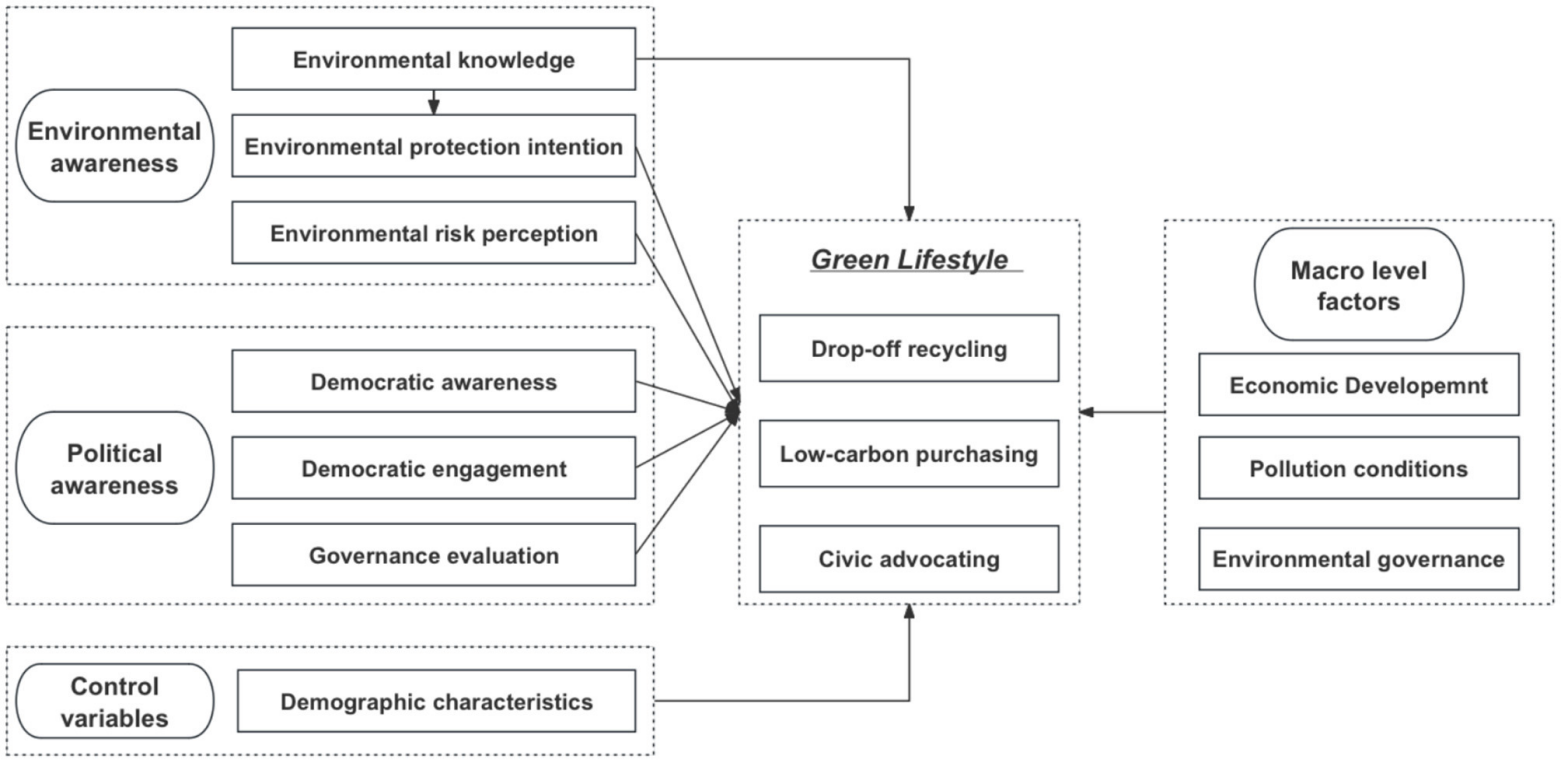
The research framework.

We suggest the research framework to analyze the factors of a GL, including three parts: drop-off recycling, low-carbon purchasing, and civic advocating (see Figure 1). Based on the norm activation theory, the theory of planned behavior, and the value-belief-norm theory, we focus on the environmental awareness module and political awareness module at the individual level. The former module includes three variables of environmental knowledge, environmental protection intention, and environmental risk perception, while the latter module consists of democratic awareness, democratic engagement, and governance evaluation. Based on the prosperity hypothesis of economic development, the driver hypothesis of environmental pollution, and the protection hypothesis of public governance, it comprises environmental governance, pollution circumstances, and economic development at the macro level.

Specifically, the present study seeks to address four research questions: (1) What is the current level of GL of Chinese residents? (2) What factors affect living a GL? (3) How do these elements affect GL through specific mechanisms? (4) Is there any difference in the intensity of the factors' influences?

## 3 Research design

### 3.1 Sample

The data utilized in this study were sourced from the 2021 Chinese General Social Survey (CGSS). The CGSS, a nationwide and comprehensive survey in China, systematically collects information from individuals aged 18 and above concerning various aspects, including social and environmental attitudes and actions. Its primary objective is to examine the characteristics and societal trends within the local context and engage in discourse on issues of both theoretical and practical significance. The survey's methodology involved a multi-stage stratified sampling procedure, wherein counties served as the primary sampling units, urban communities, and rural villages as secondary sampling units, and households were selected randomly utilizing a mapping sampling method (58).

The year 2021 was chosen as the focus due to the availability of the latest data pertaining to environmental knowledge, awareness, attitudes, and actions. Of the survey participants, approximately 33.6 percent (*N* = 8,148) were randomly assigned to respond to the environmental section, resulting in a final sample size of 2,741. We also included data on provincial environmental conditions from the 2022 China Statistical Yearbook, published by the National Bureau of Statistics of China, to match 2021 individual data with relevant contextual information. The dataset offers comprehensive insights into the evolution of the national society over the preceding calendar year.

### 3.2 Measures

#### 3.2.1 Green lifestyle

The measurement of a GL comprises three dimensions. The first is drop-off recycling, measured by how individuals intentionally sort materials such as glass, aluminum cans, plastic, or paper for recycling. The second dimension is low-carbon purchasing, measured by the frequency of individuals deliberately refraining from purchasing certain products for carbon dioxide emission reduction. Both drop-off recycling and low-carbon purchasing behaviors employed positive coding, meaning that the higher the variable values assigned, the more frequent the occurrences of GL. The third dimension is civic advocating, assessed using four binary questions in the CGSS questionnaire: “In the past five years, have you joined any environmental organizations to protect the environment, signed petitions related to environmental issues, made donations to environmental organizations, or participated in protests concerning environmental issues?” The categories were constructed as 0 = No and 1 = Yes, then, these four items were aggregated. A higher total score indicates a more frequent engagement in civic advocating within the context of a GL.

#### 3.2.2 Independent variables

##### 3.2.2.1 Environmental awareness

The environmental awareness module comprises three variables: environmental knowledge, environmental risk perception, and environmental protection intention. The environmental knowledge measurement includes ecological civilization, ecological compensation, evaluation and assessment of ecological civilization construction goals, atmospheric pollution prevention and control action plans, and more. Each item was measured into the options: “unaware (=0),” “somewhat aware (=1),” “moderately aware (=2),” “very aware (=3).” Environmental risk perception was measured by the respondents' perception of the severity of 16 types of environmental issues in their residential areas, encompassing air pollution, water pollution, soil pollution, noise pollution, biodiversity degradation, extreme weather events, resource waste, and more. Response options were structured using a 5-point Likert-type scale. For environmental protection intention, the measurement was derived from the question, “What are you willing to do to address various challenges in waste disposal?,” with the answers of recycling and reusing household items, being willing to discuss waste classification plans with other residents, proactively engaging with environmental organizations, government agencies, experts, and waste management authorities. Response options were also structured using a 5-point Likert-type scale. In the encoding process, scores for various contents within each variable were summed up so that higher scores denoted greater levels of environmental knowledge, environmental risk perception, and environmental protection intention.

##### 3.2.2.2 Political awareness

The political awareness module also consists of three variables: democratic awareness, democratic engagement, and governance evaluation. Democratic awareness was measured by the statement “The government should not interfere with individual freedom of speech, reproductive freedom, and freedom of work and life.” And the answers were coded separately using a 5-point Likert scale. Governance evaluation measured respondents' overall assessment of the central government and local government's performance in addressing environmental issues in China also using a 5-point Likert scale. Higher values for democratic awareness and governance evaluation demonstrate stronger democratic consciousness and more favorable evaluations of government governance by the respondents. The democratic engagement was measured through a binary question of whether to vote in the last neighborhood committee/city village committee election, with the value of 1 denoting actual participation and 0 revealing none.

#### 3.2.3 Macro-level variables

Three modules make up the macro-level variables. Economic development was measured using per capita regional gross domestic product (GDP). Indicators of environmental pollution include the share of the secondary industry and major air pollution (the total emissions of sulfur dioxide, nitrogen oxides, and particulate matter). Investments in pollution control and public expenditure on environmental protection are two variables of environmental governance indicators derived from the governmental fiscal spending data in the 2022 China Statistical Yearbook.

Environmental pollution indicators encompass major air pollutant emissions (total emissions of sulfur dioxide, nitrogen oxides, and particulate matter) and the share of the secondary industry. Environmental governance indicators include pollution control investments and public fiscal expenditures on environmental protection.

#### 3.2.4 Control variables

Social demographic variables selected in the analysis included gender, age, education, marriage status (coded as married and not married), political identity (coded as member of the Communist Party of China and others), ethnic group (coded as *Han* Chinese and minorities), *hukou* status (coded as urban *hukou* and rural *hukou*), and annual family income (log-transformed). We had a particular focus on media usage variables, explicitly measuring the usage of traditional media (newspapers, magazines, radio, and television) and new media (the Internet and customized mobile messages). The sum of these two constitutes total media usage.

Table 1 provides a summary of the measurements of the main variables in the current analysis.

**Table 1:** Descriptive statistics (*N* = 2,741).

	**Variables**	**Mean/ proportion**	**SD**	**Range/categories**
**Individual level**				
Environmental awareness module	Environmental knowledge	3.48	4.83	[0, 36]
	Environmental risk perception	28.34	16.64	[0, 80]
	Environmental protection intention	22.60	4.47	[6, 30]
Political awareness module	Democratic awareness	8.64	2.75	[0, 15]
	Democratic engagement	0.49	0.50	0 = not engaged, 1 = engaged
	Governance evaluation	7.20	2.32	[0, 10]
Control variables	Gender	0.46	0.50	0 = female, 1 = male
	Age	51.60	17.62	[18, 94]
	Education	9.28	4.72	[0, 19]
	Marriage status	0.70	0.46	0 = not married, 1 = married
	Political identity	0.12	0.33	0 = others, 1 = CPC
	Ethnic group	0.92	0.26	0 = minorities, 1 = *Han*
	*Hukou* status	0.41	0.49	0 = rural, 1 = urban
	Income	10.40	2.43	[0, 16.12]
	Use of new media	3.34	2.53	[0, 8]
	Use of traditional media	4.43	2.67	[0, 16]
	Use of all media	7.77	3.82	[0, 24]
**Macro level**				
Economic development module	GDP per capita	8.86	3.69	[4.10, 18.40]
Pollution module	Major air pollution	58.09	33.14	[8.89, 161.95]
	Share of the secondary industry	39.29	7.45	[18, 49.6]
Environmental governance module	Investment in pollution control	11.26	9.12	[0.635, 37.69]
	Public expenditure on environmental protection	198.90	70.45	[47.71, 351.65]

### 3.3 Analytic strategies

The primary statistical analysis is divided into four main components using Stata 17.0. Firstly, we used descriptive analysis and *t*-tests to illustrate the current state of GL among Chinese residents and to identify key demographic differences.

Secondly, utilizing multilevel linear models, we estimated the influencing factors of drop-off recycling, low-carbon purchasing, and civic advocating behaviors separately, accounting for the effects of macro-level factors. Due to the hierarchical structure of the combined data, the multilevel linear models are appropriate to partition variance at the individual and regional levels, providing insights into how contextual and individual factors influence GL while controlling for intra-regional variability. The random coefficient regressions were set up as follows:

Individual level:


$$\begin{array}{c} Y_{ik}=\delta_{0k}+\delta_{1k}X_{ik}+\delta_{z}Z_{ik}+\omega_{ik}\; \end{array}$$

Provincial level:


$$\begin{array}{c} \delta_{0k}=\sigma_{00}+\vartheta_{0k}\\ \delta_{1k}=\sigma_{10}+\vartheta_{1k}\; \end{array}$$

where ω_*ik*_ represents the individual error term.

Thirdly, employing multilevel mediational modeling methods (59), we investigated the mediating effect of environmental protection intention on the relationship between environmental knowledge and GL after controlling for macro-level factors in the clustered data. The bootstrap method is used with a 95% confidence interval (CI) to distinguish direct effects, indirect effects, and total effects.

Lastly, the sheaf coefficients analysis was conducted after multilevel linear models to compare the differential impact of core explanatory variables at both macro and micro levels. The methodology aims to directly compare impact intensities among factors based on standardized treatments (60).

## 4 Empirical results

### 4.1 The current state of GL among Chinese residents

The mean values and standard deviations for Chinese citizens' drop-off recycling, low-carbon purchasing, and civil advocacy are shown in Table 2. In sustainable practices, a discernible pattern emerges, showcasing the varying frequencies of environmentally conscious behaviors. Drop-off recycling demonstrated the most prevalent and frequent occurrence (*mean* = 2.34, *SD* = 1.09), and low-carbon purchasing followed closely but with slightly less frequency (*mean* = 2.01, *SD* = 0.97). Civic advocating was observed to be the least frequent of GL (*mean* = 0.17, *SD* = 0.49), for it involves a myriad of complex factors, encompassing public environmental inclusivity, civic behavioral norms, and civic traditions (61). This hierarchy of environmentally responsible actions reflects an intriguing spectrum of engagement with ecological concerns, highlighting the diverse approaches individuals undertake to pursue a greener, more sustainable world.

**Table 2:** Descriptive statistics of green lifestyles.

**Environmental behavior**	**Sample size**	**Mean**	**SD**	**Range**
Drop-off Recycling	2,741	2.34	1.09	[1, 4], continuous
Low-carbon Purchasing	2,741	2.01	0.97	[1, 4], continuous
Civic Advocating	2,741	0.17	0.49	[0, 4], continuous

Additionally, we looked at the demographic variations between the three eco-friendly lifestyles (see Table 3). Men had a significantly higher frequency of drop-off recycling than women (*t* = 1.92, *p* < 0.05), while significant gender disparities were not evident in the case of the other two GLs. Members of CPC were more prone to practicing GL. The mean value of drop-off recycling (*t* = 3.81, *p* < 0.001), low-carbon-purchasing (*t* = 3.37, *p* < 0.001), and civic advocating (*t* = 2.55, *p* < 0.01)were significantly higher than that of non-affiliated individuals, which might be closely connected with the CPC norms and standards. Compared with not married people, married people placed more emphasis on drop-off recycling (*t* = 3.16, *p* < 0.001) but less likely on civic advocating (*t* = 2.39, *p* < 0.01). The minorities were more likely to participate in civic advocating (*t* = 3.02, *p* < 0.01) than the *Han Chinese*.

**Table 3:** The group differences of green lifestyles.

		**Drop-off recycling**		**Low-carbon purchasing**		**Civic advocating**	
		**Mean (SD)**	***T*** **-value**	**Mean (SD)**	***T*** **-value**	**Mean (SD)**	***T*** **-value**
Gender	Female	2.30 (1.10)	1.92^*^	2.01 (0.95)	0.25	0.16 (0.47)	1.15
	Male	2.38 (1.09)		2.02 (0.98)		0.18 (0.50)	
Political identity	CPC	2.55 (1.05)	3.81^***^	2.18 (0.97)	3.37^***^	0.24 (0.58)	2.55^**^
	Others	2.31 (1.10)		1.99 (0.96)		0.16 (0.47)	
Marriage status	Married	2.38 (1.12)	3.16^***^	2.00 (0.98)	1.22	0.16 (0.47)	2.39^**^
	Not married	2.24 (1.03)		2.05 (0.93)		0.21 (0.52)	
Ethnic group	*Han* Chinese	2.34 (1.09)	1.25	2.01 (0.97)	0.03	0.16 (0.09)	3.02^**^
	Minorities	2.25 (1.10)		2.01 (0.98)		0.27 (0.62)	

Significance levels: ^+^*p* < 0.1, ^*^p < 0.05, ^**^p < 0.01, ^***^p < 0.001 (Two-tailed test).

### 4.2 Factors influencing GL

We performed multilevel models to scrutinize the interplay between macro-level and individual-level factors in shaping GL. With a specific focus on drop-off recycling (see Table 4), environmental knowledge emerged as a pivotal driver, with statistically significant coefficients ranging from 0.007 to 0.012. The correlation between environmental protection intention and drop-off recycling was strong and robust across models, with the coefficients ranging from 0.055 to 0.060. In Models 1–2, the influence of all media usage on drop-off recycling was significant (β = 0.023, *p* < 0.001). Models 1–3 displayed the negative correlation between democratic awareness and drop-off recycling in the political awareness module (β = −0.022, *p* < 0.01), and the relationship was consistent in the Models 1–7 (β = −0.021, *p* < 0.05). The influence of economic development, as represented by GDP per capita, aligned with the prosperity hypothesis, revealing a positive correlation with drop-off recycling in Models 1–4 (β = 0.056, *p* < 0.001), and the influence was still significant in the full model. Similarly, the protection hypothesis of public governance was also supported by Models 1–6 that a higher allocation of the public expenditure on environmental protection was related to more drop-off recycling (β = 0.002, *p* < 0.05). The driver hypothesis of environmental pollution was not supported by Models 1–5, since the share of the secondary industry was negatively associated with drop-off recycling (β = −0.017, *p* < 0.05 ).

**Table 4:** The multilevel models of drop-off recycling (Groups = 19).

**Variables**	**Model 1-1**	**Model 1-2**	**Model 1-3**	**Model 1-4**	**Model 1-5**	**Model 1-6**	**Model 1-7**
**Fixed effects**							
Environmental knowledge	0.012^*^ (0.005)	0.009^+^ (0.005)	0.009^+^ (0.005)	0.011^*^ (0.005)	0.012^*^ (0.005)	0.012^*^ (0.005)	0.007 (0.005)
Environmental risk perception	0.001 (0.001)	0.001 (0.001)	0.001 (0.001)	0.001 (0.001)	0.001 (0.001)	0.001 (0.001)	0.001 (0.001)
Environmental protection intention	0.060^***^ (0.005)	0.058^***^ (0.005)	0.057^***^ (0.005)	0.060^***^ (0.005)	0.060^***^ (0.005)	0.060^***^ (0.005)	0.055^***^ (0.005)
Use of all media		0.023^***^ (0.007)					0.021^**^ (0.007)
Democratic awareness			−0.022^**^ (0.008)				−0.021^*^ (0.008)
Democratic engagement			0.035 (0.048)				0.030 (0.048)
Governance evaluation			0.005 (0.011)				0.004 (0.011)
GDP per capita				0.056^***^ (0.014)			0.030^+^ (0.017)
Major air pollution					−0.001 (0.001)		−0.001 (0.002)
Share of secondary industry					−0.017^*^ (0.009)		−0.013^+^ (0.007)
Investment in pollution control						−0.006 (0.007)	0.002 (0.006)
Public expenditure on environmental protection						0.002^*^ (0.001)	0.001^*^ (0.001)
Control variables	Y	Y	Y	Y	Y	Y	Y
**Random effects**							
Variance	0.272^***^ (0.051)	0.270^***^ (0.051)	0.277^***^ (0.052)	0.185^***^ (0.042)	0.229^***^ (0.045)	0.232^***^ (0.045)	0.160^***^ (0.037)
Log-likelihood	−3,061.27	−3,055.87	−2,977.77	−3,055.82	−3,058.53	−3,058.53	−2,965.27
Wald χ^2^	186.27 ^***^	198.08 ^***^	184.86 ^***^	206.63 ^***^	194.47 ^***^	194.19 ^***^	227.64 ^***^
Observations	2,124	2,124	2,068	2,124	2,124	2,124	2,068

The cells show the coefficients and standard errors of variables. The variance component in the null model is 0.087^***^ with an Interclass Correlation Coefficient (ICC) of 0.073. Y means the variables are controlled. Significance levels: + p < 0.1, ^*^ p < 0.05, ^**^ p < 0.01, ^***^ p < 0.001 (Two-tailed test). All the results in the paper are interpreted with the same standard.

In the random effects, the models revealed consistently significant variances, ranging from 0.160 to 0.277, indicating that the variations in macro-level factors could explain about one-fifth of the provincial differences in personal drop-off recycling practices.

In the context of low-carbon purchasing (as depicted in Table 5), it demonstrated positive of new media usage on low-carbon purchasing (β = 0.028, *p* < 0.01), signifying the potential of contemporary communication platforms in fostering eco-conscious consumer behavior. Within the sphere of political awareness, a distinctive pattern emerged in contrast to our prior findings in drop-off recycling. Here, low-carbon purchasing exhibited a favorable correlation with governance evaluation (β = 0.024, *p* < 0.05). Similar to drop-off recycling, the prosperity hypothesis of economic development and the conservation hypothesis of public governance were supported as higher levels of GDP per capita (β = 0.038, *p* < 0.001) and public expenditure on environmental protection (β = 0.002, *p* < 0.01) were associated with increasing low-carbon purchasing. Except for the full model, the reduction of variance in random effects was the most in the economic development module (Models 2–4), signifying the importance of using economic conditions to explain the regional variations in low-carbon purchase behaviors.

**Table 5:** The multilevel models of low-carbon purchasing (Groups = 19).

**Variables**	**Model 2-1**	**Model 2-2**	**Model 2-3**	**Model 2-4**	**Model 2-5**	**Model 2-6**	**Model 2-7**
**Fixed effects**							
Environmental knowledge	0.019^***^ (0.004)	0.018^***^ (0.005)	0.016^**^ (0.005)	0.019^***^ (0.004)	0.019^***^ (0.004)	0.019^***^ (0.004)	0.015^**^ (0.005)
Environmental risk perception	0.004^**^ (0.001)	0.004^**^ (0.001)	0.004^**^ (0.001)	0.004^**^ (0.001)	0.004^**^ (0.001)	0.004^**^ (0.001)	0.004^**^ (0.001)
Environmental protection intention	0.059^***^ (0.005)	0.058^***^ (0.005)	0.056^***^ (0.005)	0.059^***^ (0.005)	0.059^***^ (0.005)	0.060^***^ (0.005)	0.056^***^ (0.005)
Use of new media		0.028^**^ (0.010)					0.027^**^ (0.010)
Democratic awareness			−0.000 (0.007)				0.000 (0.007)
Democratic engagement			0.053 (0.043)				0.053 (0.042)
Governance evaluation			0.024^*^ (0.009)				0.024^*^ (0.009)
GDP per capita				0.038^***^ (0.009)			−0.002 (0.029)
Major air pollution					−0.002 (0.001)		−0.002^+^ (0.000)
Share of secondary industry					−0.012^*^ (0.005)		−0.013 (0.012)
Investment in pollution control						−0.006 (0.004)	0.001 (0.003)
Public expenditure on environmental protection						0.002^**^ (0.000)	0.002^***^ (0.001)
Control variables	Y	Y	Y	Y	Y	Y	Y
**Random effects**							
Variance	0.176^***^ (0.036)	0.176^***^ (0.036)	0.175^***^ (0.036)	0.109^***^ (0.032)	0.131^***^ (0.032)	0.126^***^ (0.030)	0.040^*^ (0.036)
Log-likelihood	−2,790.07	−2,786.14	−2,714.49	−2,784.71	−2,786.26	−2,785.15	−2,697.28
Wald χ^2^	283.30^***^	292.21^***^	278.91^***^	306.37^***^	296.83^***^	300.37^***^	382.63^***^
Observations	2,124	2,124	2,068	2,124	2,124	2,124	2,068

Significance levels: ^+^*p* < 0.1, ^*^p < 0.05, ^**^p < 0.01, ^***^p < 0.001 (Two-tailed test).

Moving to civic advocating, as presented in Table 6, it is noteworthy that three variables within the environmental awareness module exhibited significant and positive correlations with increased civic advocacy. Compared to the coefficients associated with drop-off recycling and low-carbon purchasing, the coefficients relating to environmental protection intention and its impact on civic advocacy were observed to be more moderate (β = 0.012, *p* < 0.001). Traditional media usage was positively correlated to civic advocating (β = 0.010, *p* < 0.05). When considering political awareness, a higher degree of democratic engagement was significantly associated with increased civic advocacy (β = 0.057, *p* < 0.01). However, in contrast to the outcomes mentioned above, the influence of macro-level factors on civic advocacy did not yield statistically significant results.

**Table 6:** The multilevel models of civic advocating (groups = 19).

**Variables**	**Model 3-1**	**Model 3-2**	**Model 3-3**	**Model 3-4**	**Model 3-5**	**Model 3-6**	**Model 3-7**
**Fixed effects**							
Environmental knowledge	0.019^***^ (0.002)	0.018^***^ (0.002)	0.017^***^ (0.002)	0.018^***^ (0.002)	0.018^***^ (0.002)	0.018^***^ (0.002)	0.016^***^ (0.002)
Environmental risk perception	0.002^**^ (0.001)	0.002^**^ (0.001)	0.002^**^ (0.001)	0.002^**^ (0.001)	0.002^**^ (0.001)	0.002^**^ (0.001)	0.002^**^ (0.001)
Environmental protection intention	0.012^***^ (0.002)	0.012^***^ (0.002)	0.012^***^ (0.002)	0.012^***^ (0.002)	0.012^***^ (0.002)	0.012^***^ (0.002)	0.012^***^ (0.002)
Use of traditional media		0.010^*^ (0.004)					0.009^*^ (0.004)
Democratic awareness			−0.004 (0.004)				−0.003 (0.004)
Democratic engagement			0.057^**^ (0.022)				0.057^**^ (0.022)
Governance evaluation			0.007 (0.005)				−0.000 (0.005)
GDP per capita				−0.004 (0.005)			−0.012^*^ (0.005)
Major air pollution					−0.000 (0.000)		−0.001^+^ (0.000)
Share of secondary industry					0.001 (0.003)		−0.002 (0.002)
Investment in pollution control						−0.000 (0.002)	0.002 (0.002)
Public expenditure on environmental protection						−0.000 (0.000)	0.000 (0.000)
Control variables	Y	Y	Y	Y	Y	Y	Y
**Random effects**							
Variance	0.055^***^ (0.015)	0.055^***^ (0.015)	0.049^***^ (0.015)	0.054^***^ (0.015)	0.053^***^ (0.015)	0.055^***^ (0.015)	0.038^*^ (0.015)
Log-likelihood	−1,422.36	−1,419.70	−1,358.27	−1,421.97	−1,421.94	−1,422.35	−1,353.59
Wald χ^2^	179.76^***^	185.54^***^	175.18^***^	180.51^***^	180.57^***^	179.78^***^	185.77^***^
Observations	2,124	2,124	2,068	2,124	2,124	2,124	2,068

Significance levels: ^+^*p* < 0.1, ^*^p < 0.05, ^**^p < 0.01, ^***^p < 0.001 (Two-tailed test).

The random effects echoed the descriptive statistics results of three GLs. While the variances were statistically significant, the coefficients were relatively small. It suggests that a substantial portion of the variance by other relevant factors and should be taken into account in further research.

### 4.3 The mechanism of conducting GL

In our preceding regression analysis, we refrained from presenting the outcomes of introducing independent variables sequentially in order to conserve space. Actually, when these variables were systematically integrated into the model, the magnitude of environmental knowledge's influence on GL diminished after the inclusion of the environmental protection intention variable. This observation underscores the pivotal role of environmental protection intention as a significant mediating variable through which environmental knowledge exerts its influence on GL. Also, the result aligns with the propositions of the theory of planned behavior, which suggests that individuals' environmental knowledge is activated by their environmental protection intention, thereby translating into actual GL practices. The further question is whether this mechanism still holds when embedded within the influence of external environmental factors.

In contrast to conventional mediation analysis, the multilevel mediation approach directs its attention to the hierarchical structure inherent in the dataset. It not only accommodates the clustered nature of the data but also endeavors to illuminate the intricate mechanism between variables at the micro-level within the broader macro-context (59). As such, it is crucial to emphasize the specific advantages and nuances of multilevel mediation analysis and highlight its relevance in offering a more sophisticated statistical framework.

**Figure 2:**
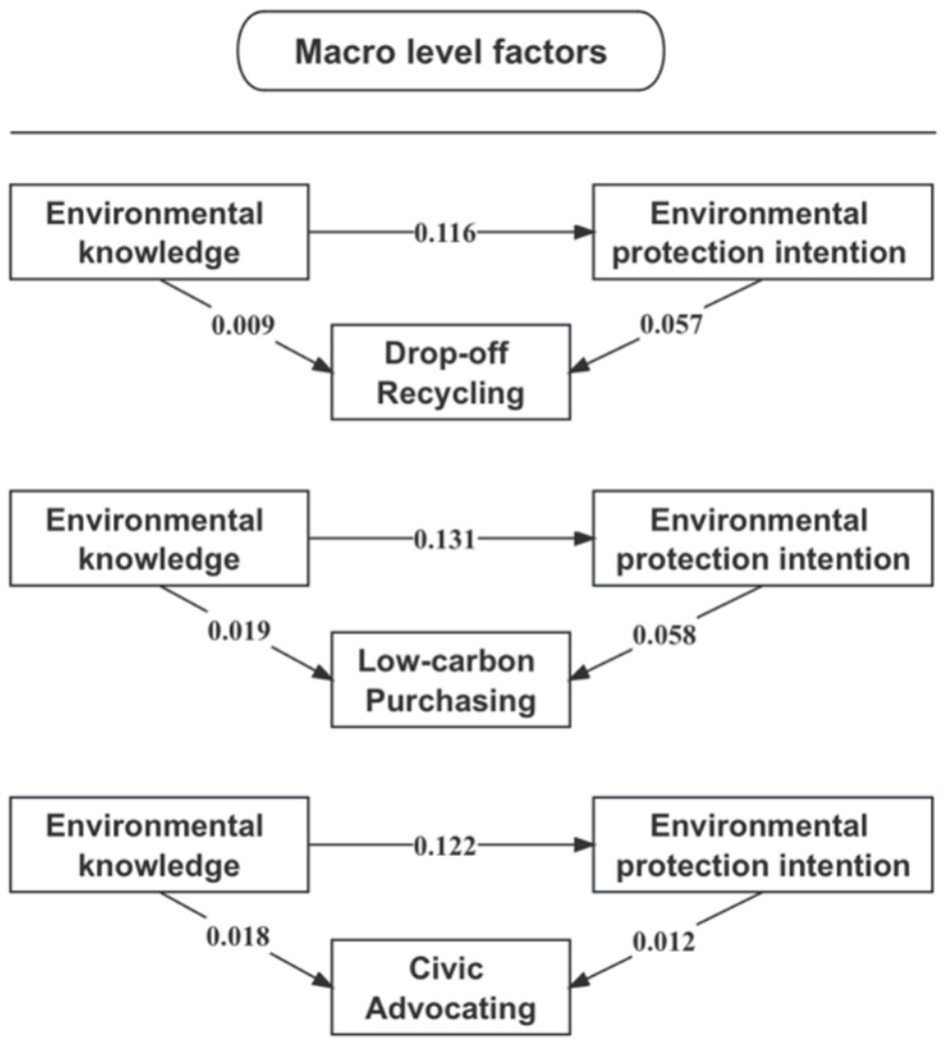
The multilevel mediation effects of environmental protection intention on green lifestyles.

The veracity of mediation was substantiated through the results of the bootstrap tests (see Table 7). The indirect effects of environmental protection intention on drop-off recycling, low-carbon purchasing, and civic advocating have all demonstrated statistical significance. The most substantial proportion of the total effects mediated pertained to drop-off recycling, accounting for 43.44%. Regarding low-carbon purchasing, 29.0% of the total effect was mediated by environmental protection intention. Civic advocacy, in comparison, exhibited a proportion of total effect mediated at 7.28%. Further insight into the coefficients of the mediational pathways can be seen in Figure 2.

**Table 7:** The multilevel mediation effects of environmental protection intention.

		**Coefficients (Standard Error)**	**Bootstrap 95%CI**		**Proportion of total effect mediated**
			**LL**	**UL**	
Drop-off Recycling	Total effect	0.015^**^ (0.006)	0.004	0.026	43.44%
	Direct effect	0.009 (0.006)	−0.002	0.020	
	Indirect effect	0.007^***^ (0.001)	0.004	0.009	
Low-carbon Purchasing	Total effect	0.026^***^ (0.004)	0.019	0.033	29.10%
	Direct effect	0.018^***^ (0.004)	0.011	0.025	
	Indirect effect	0.008^***^ (0.001)	0.005	0.010	
Civic advocating	Total effect	0.019^***^ (0.004)	0.012	0.026	7.28%
	Direct effect	0.018^***^ (0.004)	0.011	0.025	
	Indirect effect	0.001^**^ (0.000)	0.001	0.002	

Bootstrap was performed in each group with repeated sampling of 100 times. Significance levels: ^+^*p* < 0.1, ^*^p < 0.05, ^**^p < 0.01, ^***^p < 0.001 (Two-tailed test).

### 4.4 The differential impact of the core factors

We employed the sheaf coefficients clustering method to investigate disparities in the intensity of influence among the three factors contributing to GL, using calibration based on a multilevel linear model (see Table 8).

**Table 8:** The comparison of factors of green behaviors (sheaf coefficients).

		**Drop-off recycling**	**Low-carbon purchasing**	**Civic advocating**
Environmental awareness module (M1)		0.252^***^ (0.024)	0.282^***^ (0.022)	0.112^***^ (0.012)
Political awareness module (M2)		0.062^**^ (0.024)	0.061^**^ (0.021)	0.030^**^ (0.011)
Macro factors module (M3)		0.250^***^ (0.050)	0.180^***^ (0.023)	0.032^*^ (0.014)
Differences	M1 vs. M2	χ^2^ = 27.29^***^	χ^2^ = 44.19^***^	χ^2^ = 22.62^***^
	M1 vs. M3	χ^2^ = 0.00	χ^2^ = 10.04^**^	χ^2^ = 19.78^***^
	M2 vs. M3	χ^2^ = 11.78^***^	χ^2^ = 14.43^***^	χ^2^ = 0.01

Significance levels: ^+^*p* < 0.1, ^*^p < 0.05, ^**^p < 0.01, ^***^p < 0.001 (Two-tailed test).

Concerning drop-off recycling, variables within the environmental awareness module, the macro-variable module, and the political awareness module all exhibited statistically significant associations with environmentally friendly behavior. The influence of the first two modules was significantly greater than that of the political awareness module, with effect sizes of 4.06 and 4.03 times, respectively. For low-carbon purchasing, a significant difference was observed between all three modules. Environmental awareness had the most substantial impact, with an effect size of 4.62 times that of political awareness. For civic advocating, the coefficients of influence from each module exhibited a reduction in comparison to the other aspects of GL. The difference between the influence of macro variables and political awareness was not statistically significant (χ^2^ = 0.01, *p*>0.05 ).

Among the factors shaping environmental behavior, environmental awareness yielded the most potent influence, while political awareness had the weakest impact. The macro-environment exerted more influence on drop-off recycling and low-carbon purchasing compared to civic advocating.

## 5 Discussion

This study, grounded in the persistent contradiction between ongoing economic development and escalating environmental pollution, scrutinized the influencing factors on residents' GL at micro and macro levels, elucidated the functioning mechanisms, and assessed the relative strengths of these influences, all aimed at augmenting people's adoption of a GL. The key findings of this research can be summarized as follows.

Firstly, residents exhibited a moderately low level of GL practices, with civic advocating being notably limited, considerably lower than those in Europe and Japan, where people have multiple carbon footprint reduction potential of lifestyle choices (62, 63). The members of the CPC demonstrated significantly higher levels of engagement across the three GL dimensions compared to non-party members, whose discrepancy can be attributed to income and educational attainment, along with social capital and network.

Secondly, environmental knowledge and environmental protection intentions consistently displayed significant effects among the micro-level factors influencing residents' GL. When controlling for macro-level factors, converting environmental knowledge into tangible green behaviors primarily hinged on elevating environmental protection intentions. While a meta-analysis focusing on international research in 2004–2014 finds that in developed and individualistic countries, environmental protection intentions are more likely to translate into actual behavior (43), the current study provides evidence for developing and collectivist countries.

Education offered an institutional framework (39) within which individuals were inspired by environmental knowledge to expand the scale and quality of GL. Media utilization served as a non-institutional means (40). It showed variations in shaping different forms of GL: new media increased low-carbon purchasing, while traditional media promoted engagement in civic advocating. Media propaganda fostered rational and scientific perceptions of environmental issues and events and disseminated information from non-governmental organizations. It would be beneficial to call for green consumption behaviors through new media while capitalizing on the expertise and credibility of traditional media to establish public participation channels. As for the influence of political awareness, its impact was multifaceted, reflecting the nascent stage of political democratic awareness and behavior among the Chinese population. Unlike liberalism's positive effect on green purchase behavior and political engagement in socio-ecological issues (13), conservative attitudes and reliance on government intervention rendered their effect on GL rather subtle.

Thirdly, within macro-level factors, the significantly positive impact of financial expenditures and economic development levels underscored the need to strengthen environmental governance, promote social development, and fully leverage government fiscal protective roles. Nevertheless, it is imperative to assess the extent of government involvement in environmental protection endeavors to circumvent potential infringements on individual agencies within the public domain.

We acknowledge several limitations of the study. Firstly, the discussion of macro-level factors and their internal linkages could be more extensive to explore potential macro-micro moderating effects. Additional pathways such as social norms and post-materialism ideology could assist in a deeper understanding of the mechanism underlying GL. A richer and more nuanced framework including moderating and mediating effects would provide a more holistic perspective on GL. Secondly, our study was limited to establishing correlational relationships rather than causal analyses. Future studies could build on the findings by leveraging longitudinal datasets when they become accessible. The cross-sectional data in the present study lack a temporal dimension and therefore cannot analyze the dynamic relationships between variables over time, which is a key strength of random effects models in longitudinal data. Thirdly, we believe that seeking the spatiotemporal patterns of GL within the country or internationally is a promising field of research and is worthy of further analysis. It would capture more cultural, institutional, and social differences and expand the applicability of our findings in China. Moreover, we must approach the results derived from post-COVID-19 data with caution. The pandemic likely caused both temporary and long-term shifts in behavior, such as a reduction in carbon footprints, disruption of social norms, and rise of digital and remote behaviors. Future studies could explore these impacts by comparing pre- and post-pandemic data to understand how GL has evolved in response to the pandemic.

There is a need for targeted environmental education campaigns to improve environmental knowledge and foster green lifestyles at the individual level with the assistance of NGOs, public welfare organizations, and online communities. Meanwhile, recognizing the differential impact of traditional and new media on civic engagement and daily practices, policymakers should leverage both forms of media and establish partnerships with media outlets to enhance the power of media influence. They can form partnerships with Internet influencers to normalize sustainable consumption behaviors among younger generations and increase online green communities. As for the macro-level implications, policymakers should prioritize economic diversification and allocate sufficient funds to environmental protection initiatives, including optimizing green taxation and subsidies, adopting region-specific sustainable strategies, promoting innovative carbon-reduction technologies, reducing reliance on secondary industry, and fostering a transition for a greener economy. Future studies might explore how specific green policies, such as waste sorting and recycling initiatives, influence residents' GL across provinces and districts. It would address gaps in spatiotemporal patterns or causal analysis by incorporating fine-grained spatial and longitudinal data.

## Data Availability

The original contributions presented in the study are included in the article/supplementary material, further inquiries can be directed to the corresponding author.
